# Comprehensive Characterization of Polymeric Composites Reinforced with Silica Microparticles Using Leftover Materials of Fused Filament Fabrication 3D Printing

**DOI:** 10.3390/polym13152423

**Published:** 2021-07-23

**Authors:** Waleed Ahmed, Sidra Siraj, Ali H. Al-Marzouqi

**Affiliations:** 1 Engineering Requirements Unit, COE, United Arab Emirates University, Al Ain 15551, United Arab Emirates; 2 Chemical Engineering Department, COE, United Arab Emirates University, Al Ain 15551, United Arab Emirates; sidra.siraj@uaeu.ac.ae (S.S.); hassana@uaeu.ac.ae (A.H.A.-M.)

**Keywords:** polymers, silica, nylon, waste, 3D printing

## Abstract

Silica exhibits properties such that its addition into polymeric materials can result in an enhanced overall quality and improved characteristics and as a result silica has been widely used as a filler material for improving the rheological properties of polymeric materials. The usage of polymers in three-dimensional printing technology has grown exponentially, which has increased the amount of waste produced during this process. Several polymers, such as polypropylene (PP), polyvinyl alcohol (PVA), polylactic acid (PLA), and nylon, are applied in this emerging technology. In this study, the effect of the addition of silica as a filler on the mechanical, thermal, and bulk density properties of the composites prepared from the aforementioned polymeric waste was studied. In addition, the morphology of the composite materials was characterized via scanning electron microscopy. The composite samples were prepared with various silica concentrations using a twin extruder followed by hot compression. Generally, the addition of silica increased the tensile strength of the polymers. For instance, the tensile strength of PVA with 5 wt% filler increased by 76 MPa, whereas those of PP and PLA with 10 wt% filler increased by 7.15 and 121.03 MPa, respectively. The crystallinity of the prepared composite samples ranged from 14% to 35%, which is expected in a composite system. Morphological analysis revealed the random dispersion of silica particles and agglomerate formation at high silica concentrations. The bulk density of the samples decreased with increased amount of filler addition. The addition of silica influenced the changes in the characteristics of the polymeric materials. Furthermore, the properties presented in this study can be used to further study the engineering design, transportation, and production processes, promoting the recycling and reuse of such waste in the same technology with the desired properties.

## 1. Introduction

**Figure 1:**
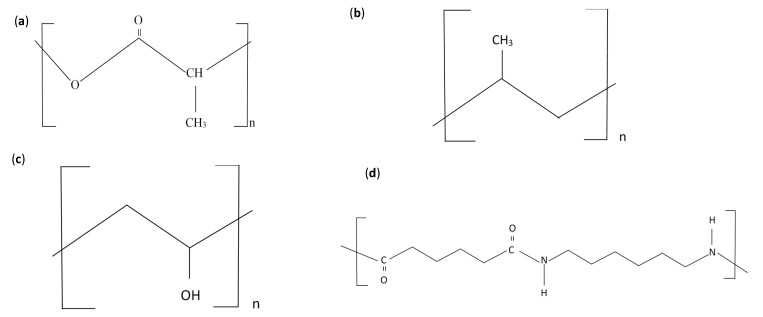
Polymer structures of (**a**) PLA, (**b**) PP, (**c**) PVA, and (**d**) nylon 6,6.

The global plastic consumption has drastically increased during the previous century, considerably increasing the amount of generated plastic waste [1]. Researchers worldwide have been attempting to develop sustainable solutions to address the increase in plastic waste and preserve the environment [2]. Among the commonly used polymeric materials, high-density polyethylene (HDPE), polypropylene (PP), and nylon are widely used in industries ranging from domestic packaging to automotive applications, primarily to enhance the mechanical properties of materials [3,4,5,6,7]. In addition, among bio-based polymers, polylactic acid (PLA) and polyvinyl alcohol (PVA) are widely preferred in applications involving hydrogels, catalysts, and strengthened materials. They are even used in rapid manufacturing and prototyping to develop products such as packaging applications [8,9,10,11]. Figure 1 shows the polymeric long-chain molecular structures of PLA, PP, PVA, and nylon. 

The recycling of polymeric waste materials is being increasingly studied to achieve a sustainable approach [12,13,14]. Innovative techniques have also been developed to convert thermoset plastics into thermoplastic ones to improve their recycling [15,16]. Compared with other waste disposal techniques, recycling is considered a more economical and practical approach. However, mechanical recycling is limited because of the toxic compounds associated with recycling, even though it is a highly resource-effective approach. The three-dimensional (3D) printing technology has emerged as the latest sustainable technique to promote the use plastic waste as feedstock material [1,17,18].

Commercial PLA degrades at a slower rate under natural environmental conditions, resulting in its prolonged existence [19]. In addition, PLA disposal results in the loss of several substantial hydroxyl components that can be effectively used as monomers. Therefore, PLA should be recycled to extract and reutilize such valuable compounds that can be used to develop various monomers [20]. Moreover, PLA can be easily separated from other polymers because of its higher density [9]. 

Polypropylene (PP), which is extensively used as an industrial-scale commodity polymer, is another polymer that has drawn considerable attention because of its numerous advantages, including its lightweight nature, high moisture resistance, chemical stability, and ease of processing [21]. PP has a high melting temperature and exhibits superior mechanical and electrical characteristics compared with other polymers [22,23]. It is also considered an environmentally friendly insulation material and power capacitor. However, its direct manufacturing usage is limited because of its low-temperature impact strength [24].

PVA is well known as the largest synthetically produced polymer worldwide [25]. PVA is the chosen polymer for many biochemical, pharmaceutical, and biological applications because of its high chemical resistance, outstanding water solubility and biodegradability [26]. The ability of PVA to be produced as a hydrogel makes it adaptable to complex medical applications such as synthetic organ (ex; heart) production [8]. Even though PVA exhibits good stability, it provides no essential benefits as a polymer itself. However, because it functions as a good host, it can be used as an excellent carrier to deposit valuable compounds to the required sites for realizing enhanced properties. Hence, it is widely preferred to be used in polymer composite materials as a matrix [27]. 

The polymers in synthetic fibres, such as nylon, have also been used to develop rubbers and resins [28]. In addition, fibre–rubber composite mixes have drawn considerable attention because they exhibit better properties than pure fibre-based materials. For example, the addition of fillers to fibres can result in improved mechanical strength and surface wettability. Therefore, recent studies have focused on fibre composites [29]. 

As mentioned earlier, the utilization of pure polymeric materials is not preferred because of their inferior properties compared with those of polymer composite materials containing a weighted percentage of filler or particulate matter [30,31,32]. A study with talc filler addition promoted this idea as well for thin sheet applications [33]. The addition of fillers results in enhanced mechanical and thermal properties and improves polymers’ conductivity and barrier properties. Therefore, polymer composite materials are preferred over pure polymers in most industrial applications such as packaging material [34,35,36,37]. Inorganic fillers, such as calcium carbonate (CaCO_3_), titania (TiO_2_), silica (SiO_2_), and graphene, have been used as fillers [38,39,40,41]. Among the commonly used fillers, silica is considered an excellent choice for various applications because of its highly unreactive nature, high chemical and thermal stability, and the large number of silanol groups that can be easily functionalized [42]. Silica has thus been widely used as a filler in reinforced building materials and improves the thermal and mechanical properties of composites [7,43,44,45]. Because silica exhibits excellent properties, using silica as a filler has emerged as an interesting research field.

In general, fused filament fabrication (FFF), also known as fused deposition modelling (FDM) or more commonly well-known as 3D printing, is one of the most traditional, affordable and easy to use 3D printing technologies. Although there are many alternative meanings for 3D printing technology, according to ISO/ASTM 52900:2015—Additive manufacturing—General principles—Terminology, 3D printing is defined as: “[the] *fabrication of objects through the deposition of a material using a print head, nozzle, or another printer technology*” while additive manufacturing (AM) is “[the] *process of joining materials to make parts from 3D model data, usually layer upon layer*”. 3D printing offers low cost, low energy consumption, and simple modelling techniques specifically for small intricate parts, making it an attractive fabrication technique [1,18]. Although the advances in 3D printing have led to growth in rapid testing and prototyping, which has resulted in the spread of knowledge across fields, the exponential rise of 3D printing has also resulted in increased waste production [36,37,38]. Fortunately, 3D printing can also be performed using waste raw materials, effectively conserving energy. A more centralized recycling approach could benefit from the usage of 3D printing technology because household waste constitutes approximately 31% of the residual waste [1,46]. Moreover, the waste of 3D printed composite material can also be easily recycled, making this method an eco-friendly disposal method as well for a wide range of composites [37].

Continuously produced reinforced fibres have already been studied [47]. The incorporation of silica particles into polymers, such as PLA, PP, PVA, and nylon, for 3D printing, enhances the material characteristics [48,49]. For instance, PLA-/silica-based medical-grade scaffolds have been developed using the 3D printing technology [35,50]. PVA-/silica-based fibres have also been incorporated with 3D printing to develop drug delivery patches [51]. Moreover, it has been demonstrated that the addition of nano-silica in PP resulted in the improvement of foam quality—as assessed from the well-defined and regular cell structures with absence of cell coalescence—as well as an increase in expansion ratio and decrease in foam density [52], and that significant improvements in the thermal stability were noticed with an increase in the percentage of nanosilica particles whereas the mechanical properties were improved [53], especially polypropylene modified by blending elastomer and nano-silica as the dispersed polyolefin elastomer could transfer and homogenize external mechanical forces [54], but for the electrical properties, when nano-SiO_2_ was more dispersed in the PP phase, the space charge improvement effect was best [55].

Functionally graded materials (FGMs), such as the functionally graded nylon, involve the selected transitions concerning the material properties for enhancing the functional value of the corresponding component. Although FGMs are widely used in several engineering applications, the development of complicated parts from FGMs has been limited. However, the effective usage of nylon in 3D applications has been promoted in emerging research [56,57], enabling the potential for reutilizing the produced wastes using 3D techniques. Inevitably, reusing polymeric waste as a feedstock material is an added advantage associated with 3D printing applications [11,58]. Fused filament fabrication (FFF) is considered a simple and nonexpesive additive manufacturing technology that has unlimited applications [59,60,61], especially when using recycled plastics that could be further enhanced with different fillers. Silica particles are considered superior alternative fillers for improving the properties of polymers [62]. The literature also supports using in situ-synthesized silica particles to develop composite materials with improved properties [63,64].

In this study, polymeric waste obtained from the 3D printing of four different polymers (PP, PVA, PLA, and nylon) was collected to study the effect of the addition of silica on the mechanical and thermal properties of the developed materials. This study can help us understand the difference between the properties of commonly used polymers when considering identical particle reinforcements. In addition, this study promotes the idea of reusing composite materials in a similar 3D printing application. 

## 2. Materials and Methods

Assorted leftover polymers (PLA, PP, PVA, and nylon) from the 3D prototyping laboratories of the United Arab Emirates University (UAEU) were collected, sorted, and shredded using a DIY shredder machine [65]. Notably, the shredded material contained no residue [66] because the plastic waste of the university laboratory was carefully sorted. Silica, which was collected from the local environment, was cleaned, sieved, and ground using a heavy-duty grinder (planetary ball mill, PL-400, Retsch, Germany). The detailed grinding process and composition of the sand sample are described elsewhere [67]. 

**Figure 2:**
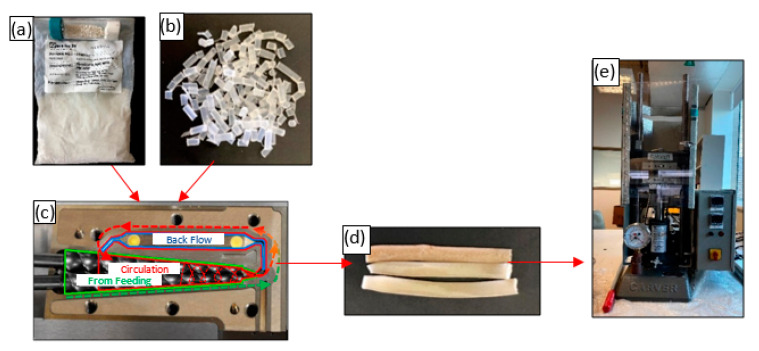
Material preparation: (**a**) powdered silica, (**b**) polymer sample, (**c**) twin-screw extruder (top view), (**d**) extruded filament, and (**e**) compression molding machine.

The polymeric waste samples were cut and further shredded using a mechanical shredder. The shredded particles were approximately 1 mm in length, which was sufficiently small for them to fit through the feed hopper of the extruder. In this study, various mixing ratios (5, 10, and 15 wt%) of the final powdered form of silica (<5 µm) were mixed with each set of polymeric waste. The polymer and microparticles were melt-blended into a composite material in a twin-screw extruder (MiniLab Rheomex CTW5, HAAKE, Karlsruhe, Germany). The melt blending procedure followed a closed-loop cycle set for approximately 5 min at 190 °C with a rotational speed of 140 rpm. The same procedure was performed for each set of polymers alone (i.e., 0 wt% silica). After the closed-loop cycle, the blended material was extruded through a valve at the exit of the extruder to collect the composite material for the compression molding step. In this stage, approximately 1 g of the extruded sample was chopped and thermally compressed using a Carver’s press (Carver^TM^ Lab Presses, IN 46992-0554 USA) under 5000 psi and at the same extrusion temperature and time set (i.e., 190 °C and 5 min). This process was performed for the extruded samples obtained from each polymer with the desired filling ratio. The samples obtained from this process were thin sheets of the composite material investigated for their mechanical and thermal characteristics. Figure 2 shows the material preparation, extrusion, and compression processes.

### 2.1. Mechanical Characterization

Different mechanical properties of the materials were tested using a universal testing machine (UTM, Shimadzu, Kyoto, Japan) to study the effect of silica reinforcement on the polymer/composite samples. The thin composite sheets obtained from the compression molding stage were used to prepare the tensile test samples following the American Standard Society for Testing and Materials (ASTM)-D 638 norms. The specific dimensions were achieved using a specialized manual blanking machine (Exacta Model-JFP, Bengaluru, India).

A common problem observed during the cutting process is that the clearance between the die and the punch must be a function of the thickness of the sample because the clearance represents the ratio to the thickness of the sample [68,69]. Consequently, the sheared edges might be affected, resulting in forming of rough surfaces, which affect the sample’s mechanical properties and could contribute to inconsistent failure. Generally, the stress concentrations at the ends of the sample can affect the mechanical behaviour of the sheets. The pressure created on the punch and die during the blanking process can lead to stress distributions, which can influence the difference in geometry at a given burr size and the surface of the intersection [70].

**Figure 3:**
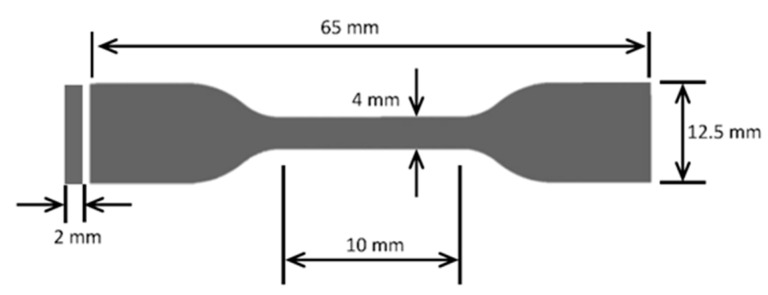
Dimensions of one of the prepared samples.

**Figure 4:**
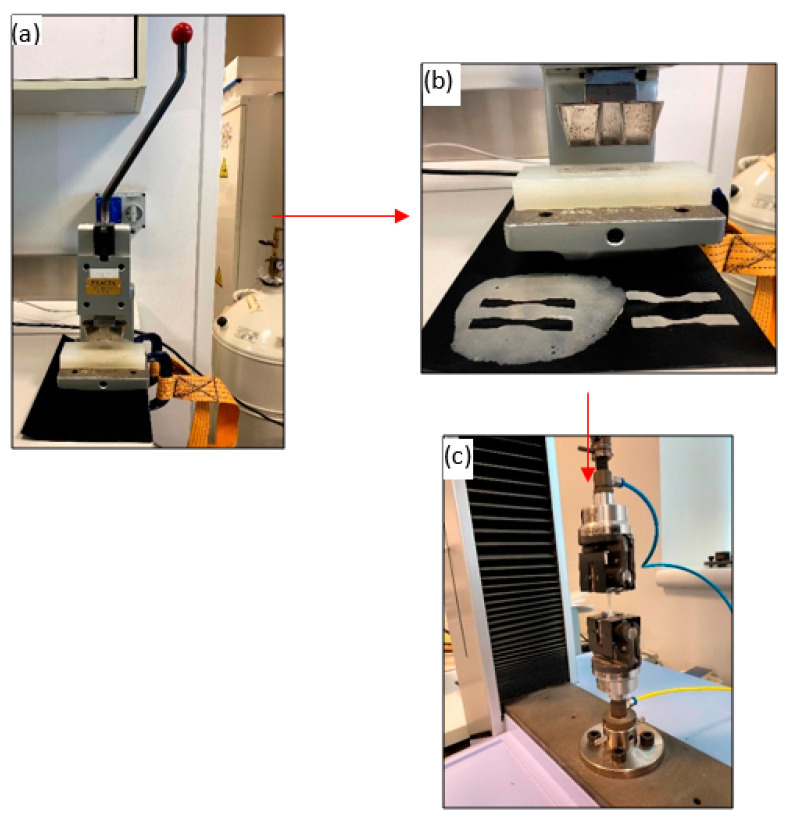
(**a**) Blanking machine; (**b**) Dumbbell shape samples after blanking process (**c**) Tensile testing machine with a sample.

Dumbbell-shaped tensile test specimens were prepared using the blanking machine. The dimensions (i.e., the length (l), width (w), and thickness (t)) of each set of the composite samples are presented in Table 1. The varying thicknesses of the pieces can be directly attributed to the processing of the polymers themselves. The corresponding mechanical properties were investigated using a 10 kN load cell with a 5-mm/min crosshead motion. Five replicates measurements were performed for obtaining increased accuracy, which is represented as standard deviations (SDs) in the plots of the results. Figure 3 shows the dimensions of one of the tensile test specimens, and Figure 4 shows the blanking process and the tensile testing machine.

### 2.2. Differential Scanning Calorimetry (DSC) 

The melting temperatures (*Tm*) and crystallization temperatures (*Tc*) of the control samples, i.e., the pure polymeric sheets and the prepared composite sheets, were determined using modulated differential scanning calorimetry (Discovery DSC 25, TA Instruments, New Castle, DE, USA). Samples of approximately 5–10 mg were heated from 20 to 200 °C at a heating rate of 40 °C/min to remove the thermal history of the polymer composite material. After the thermal history was eliminated, all the samples were cooled from 200 °C to room temperature (20 °C) at 10 °C/min for recording Tc and subsequently heated from 20 °C to 200 °C at 10 °C/min to record Tm. All the experiments were carried out under an inert flowing N_2_ atmosphere. 

### 2.3. Scanning Electron Microscopy (SEM)

A scanning electron microscope (JSM 6390A, JEOL, Peabody, MA, USA) operated at 2 kV with a spot size of 40 was used to image the prepared composite sheet materials. The composite sheets were placed on an Al pin mount adapter using double-sided carbon tape. They were then sputter-coated with Au (Au/C) using a sputter coater under vacuum to avoid electrostatic charging during the examination. This was also done to improve the conductivity of the sample and enhance the quality of the images. The scanning electron microscope was operated in high-vacuum mode with an acceleration voltage of 15 kV, and images were acquired. The working distance for the SEM was 1.5 mm

## 3. Results and Discussion

### 3.1. Mechanical Characterization

The developed composite sheet material was used to prepare dumbbell-shaped specimens for mechanical testing. Several samples were prepared from each specimen so that the tests could be repeated, improving the test’s overall accuracy. A thickness gauge (model 547–526S, Mitutoyo, Kawasaki, Japan) with a resolution of 0.001 mm was used to determine the thickness of the samples at various locations along the gage length. The samples exhibiting consistent values were selected for further testing [71]. 

**Figure 5:**
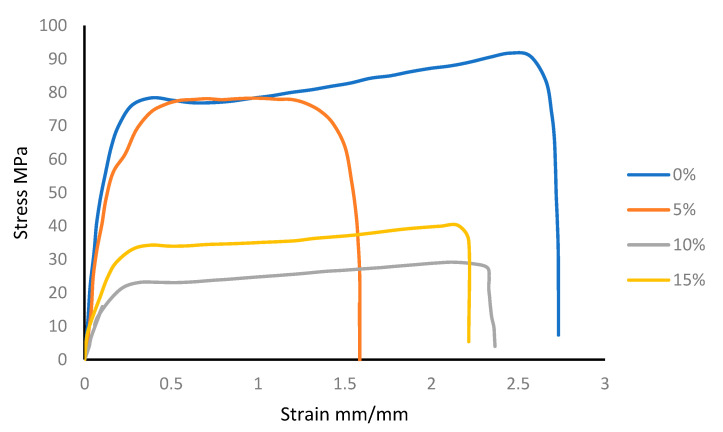
Nylon/silica stress-strain behavior.

**Figure 6:**
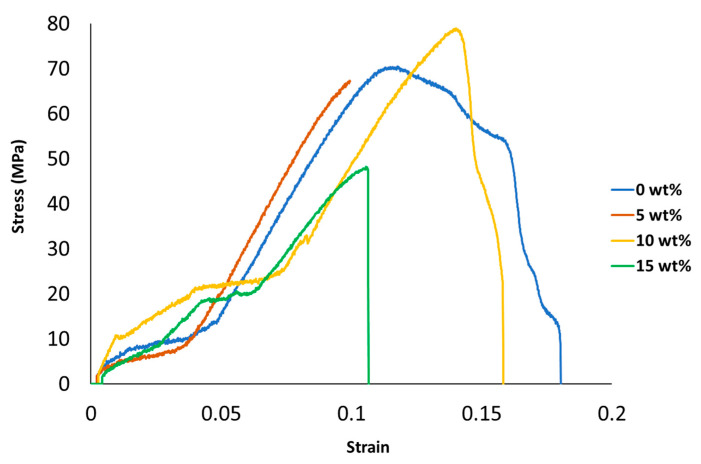
PLA/silica stress-strain behavior.

Figure 5 shows the the stress-strain curves of nylon mixtures, whereas in Figure 6 the stress-strain curves of the PLA blends are illustrated.

**Figure 7:**
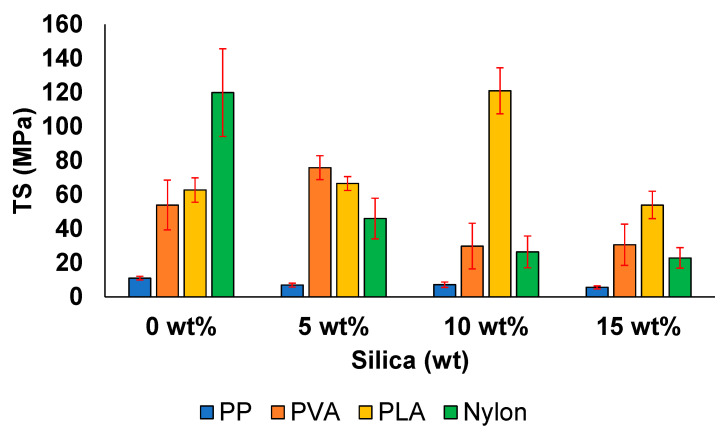
Tensile strength values of the prepared samples.

Figure 7 shows that the TS of the samples increases with the addition of silica particles. In case of PP, the addition of silica results in a gradual decrease of the TS from 11.01 MPa at 0 wt%, which is similar to the value of 5.63 MPa obtained at 15 wt% mentioned in the literature [72]. However, the TS value slightly increases from 6.96 MPa at 5 wt% to 7.15 MPa at 10 wt%. This behaviour of PP has been previously reported to occur at a silica content of approximately 3 wt%. This behaviour is likely to be associated with the stiff layers of silicate, which have a high aspect ratio, causing a high degree of interaction and suitable interfacial adhesion property. In addition, this behaviour contributes to the restriction of the free mobility of the polymer chains, increasing the TS value [72,73]. 

In the case of PVA, the TS value was observed to drastically increase from 54 to 76 MPa with the addition of 5 wt% silica, indicating enhanced mechanical properties. The TS value decreased when considering higher silica contents. Similar mechanical values have been previously reported; for example, a TS value of 71.1 MPa has been obtained for pure PVA [74]. The sharp rise in tensile strength can be related to the co-condensation observed at this composition, resulting in the increased uniformity and dispersion of the filler. However, the decrease in TS value at other filler compositions is associated with weak interactions between the matrix and filler [8].

In case of PLA, the TS value considerably decreased with the increasing silica concentration until the silica concentration reached 10 wt%. This increase in the TS value directly corresponds to the positive effect of the addition of a filler into the matrix. At higher silica concentrations, the TS decreased to become 54 MPa, similar to the results of previous reports [67]. Similar values have been reported for PLA. However, beyond a specific weight concentration of filler, the mechanical properties tend to deteriorate because the lack of consistent mixing of particles contributes to stress concentration and results in failure [44].

In case of nylon, the TS value gradually decreased from 120 MPa at 0 wt% to 23 MPa at 15 wt%. This is because the weak interfacial bonding caused by micro fillers results in the increased brittleness of the matrix, reducing the strength of the material [57]. 

The decrease in the TS value of all the samples can be attributed to the random dispersion of the filler inside the polymeric matrix. This can result in the direct agglomeration of particles beyond a filler content of 0 wt% or beyond the absolute weight concentration of the filler, which the polymer can withstand. This agglomeration results in defects and creates initiation spots that can lead to failure. Voids are commonly observed to form in polymer composite systems, adversely affecting their mechanical behaviour. In addition, the irregular shapes of the particles make them unable to uniformly transfer stress, resulting in diminished mechanical properties [72,73,75]. 

**Figure 8:**
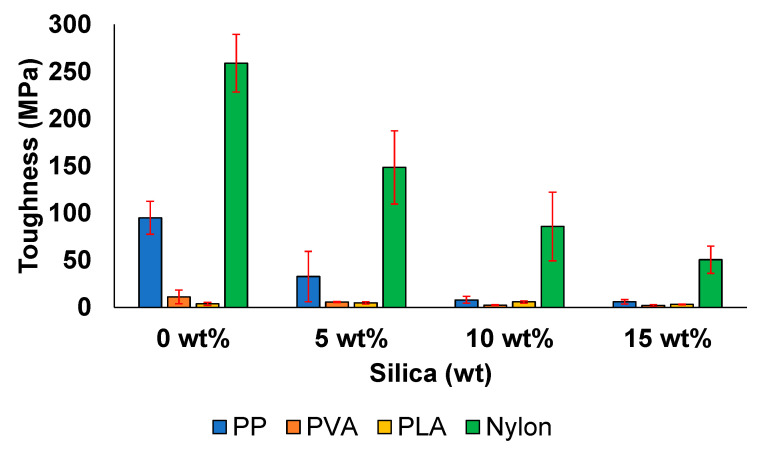
Toughness of the prepared composite materials.

Figure 8 presents the toughness of the prepared composite materials. The toughness values of the PP and nylon samples are considerably greater than those of the PVA and PLA samples. The toughness, which is a measure for absorbing energy, decreased from 95 MPa at 0 wt% silica to 6 MPa at 15 wt% silica in case of PP. This decrease can be attributed to the agglomerates that affect the structural stability of the prepared composite materials, reducing the absorption of impact energy by the material and inducing crack formation at the interface of the material. To address this issue, several researchers have incorporated modifying agents that improve the impact resistance of materials [72,76].

The toughness value of PVA decreased by 82% (from 11 to 2 MPa) when silica content was increased from 0 to 15 wt%. Such a substantial decrease in toughness has been observed in other PVA composite systems and can be attributed to the adsorption of the silica clusters between PVA molecular chains [8,26].

The toughness of the PLA/silica samples increased from 3.6 to 5.6 MPa when the silica concentration was increased from 0 to 10 wt%, implying the positive effect of the addition of 10 wt% filler on the toughness of the prepared sample, which is in agreement with the literature [67]. This improved toughness value corresponds to enhanced energy absorption ability. Enhanced toughening in the case of the addition of 10 wt% silica has also been reported in a previous study [77]. In addition, this behaviour can occur because the silica particles promote only partial stress within the PLA matrix, changing the direction of crack development [78,79,80]. The increase in toughness value until the filler concentration reaches a certain level (in this case, 15 wt% silica) followed by a decrease when the filler concentration is increased further can be explained based on the deboning mechanism. In this mechanism, voids are created between the silica/PLA interface, leading to shear yielding in the polymer matrix, which can only toughen the material to a certain level. As a result, agglomeration occurs beyond a certain filler content, resulting in defect points that adversely affect the mechanical properties [42,77]. The concentration and particle size of the filler can strongly contribute to the changes in mechanical properties, as reported in other studies of PLA/silica [81,82].

Nylon exhibited the largest decrease in toughness value, from 259 MPa at 0 wt% to 50 MPa at 15 wt%. In addition to weak bonding at higher filler concentrations, the substantial increase in energy density owing to the addition of a filler, which can lead to the rapid degradation of the polymer, likely contributed to the decrease in the TS and toughness of the material [57].

**Figure 9:**
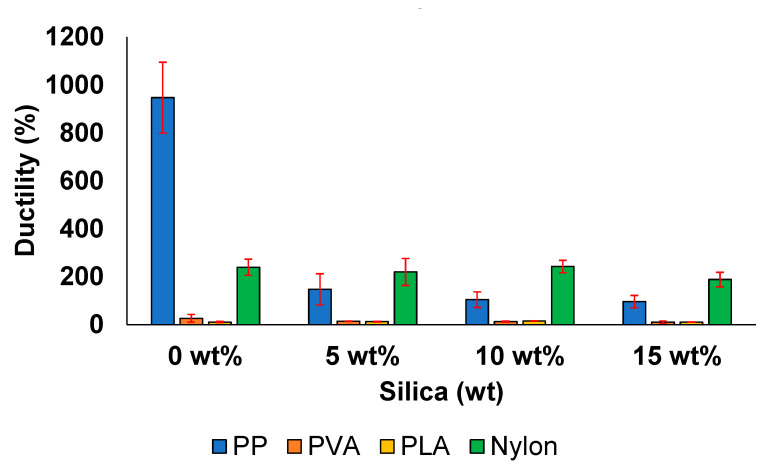
Ductility of the prepared samples.

The ductility of the material indicates the strain at the point of failure. The ability to deform plastically and adapt to the applied load is attractive in industrial applications that require high flexibility. Similar to the trend observed in the case of toughness, PP and nylon exhibit ductility values that are relatively greater than those of PVA and PLA (Figure 9). 

In case of PP/silica samples, ductility decreased considerably (90%) when the silica concentration was increased from 0 to 15 wt%; the samples demonstrated a final elongation of 96%. Further, the degradation behaviour is well associated with the development of structural voids and unexfoliated aggregates, reducing the ability of the matrix to exhibit enhanced ductility, as reported in other studies conducted on PP matrix systems [72]. 

A typical decrease in ductility values from 16 MPa at 0 wt% to 10 MPa at 15 wt% was observed in the prepared PVA/silica samples. Such a classic mechanical trend can be attributed to the non-homogeneous dispersion of the micro-sized silica in the polymer matrix and has been well documented in similar PVA/silica composite studies [8].

The ductility of the PLA sample prepared with 5 wt% silica increased by 13%, whereas an increase of approximately 11% was reported previously [83]. Moreover, the sample exhibited the highest ductility (37.5%) when considering the addition of 10 wt% filler, which corresponded to an elongation of 15.3% [67]. The ductility decreased by 2.1% at higher silica concentrations. The lower ductility values are reflected again by the stress concentrations at different locations because of the weakening effect of the agglomerates formed in the matrix [3].

Similar to the PP and PVA samples, the nylon samples exhibited a steady decrease in ductility with the increasing silica concentration. The ductility of the 0 wt% silica sample was 239%, whereas that of the sample with 15 wt% silica was 188%. This trend has also been observed in other studies [29]. The combination of a ductile polymer with short, brittle particles or fibers has been reported to result in lower strain values at the failure point compared with the strain values of neat polymers. 

**Figure 10:**
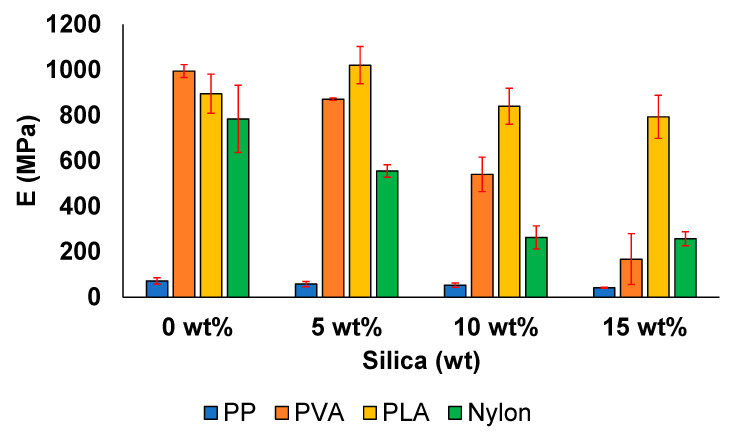
Elastic modulus of the prepared samples.

**Figure 11:**
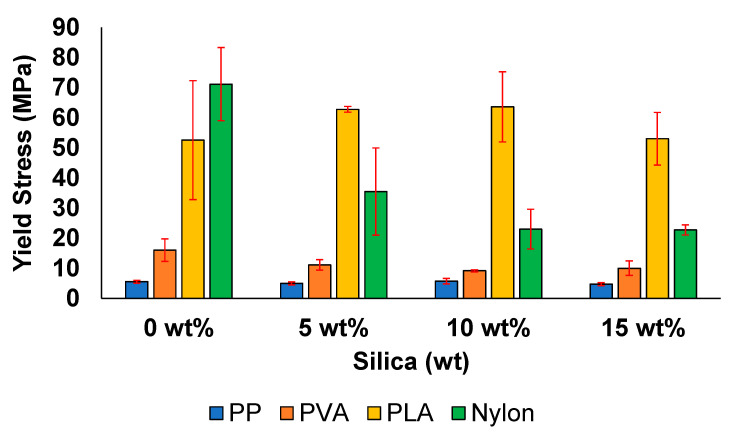
Yield stress of the prepared samples.

The elastic modulus (E) and yield stress associated with all the samples are shown in Figure 10 and Figure 11. In the case of PP, E gradually decreased from 72 MPa at 0 wt% to 41 MPa at 15 wt%. A similar behaviour was observed for its yield stress values (Figure 11). This decrease in mechanical property with the increasing filler amount is associated with the agglomerates in the filler matrix. Thus, stress concentrations are induced at several locations in the composite material, reducing the filler-aspect ratio. This further decreases the area available for forming a contact surface between the filler and matrix, leading to the formation of tactoids [72]. 

All the other samples exhibit a relatively higher E value and yield stress when compared with the samples prepared with PP. For instance, in case of PVA, the E values and yield stress decreased from 995 and 16 MPa at 0 wt% to 167 and 10 MPa at 15 wt%, respectively. The inability to form stronger bonds with the addition of the filler leads to a decrease in the E value with the increasing silica content, which has also been previously reported [27].

In the case of the PLA, a noticeable increase was observed in the elastic modulus from 895 MPa at 0 wt% to 1021 MPa at 5 wt%. The elastic modulus decreased at filler concentrations greater than 5 wt%. In addition, one of the highest yield stress values was observed at the same filler concentration, i.e., approximately 63 MPa, which is at least 21% higher than the value reported for pure commercial PLA (49.5 MPa) [83]. The increase in the elastic modulus and yield stress values are positive effects of filler addition [67]. However, the decrease in both properties at higher silica concentrations is related to the agglomerate formation and uneven crystallization, resulting in weak bond formation. 

The samples prepared with pure nylon exhibited the highest E and yield stress values among the samples investigated in this study. However, a decreasing trend in both mechanical properties was obtained when the filler concentration was increased from 0 to 15 wt%. A reduction of almost 67% was observed with respect to both the elastic modulus and the yield stress value when the filler concentration was increased from 0 to 15 wt%. This behaviour has been previously reported for nylon samples with silica concentrations from 2 to 10 wt%, which has been attributed to the increasing stiffness and brittleness of the samples [56]. 

The mechanical properties of the prepared samples, except for the samples prepared with PLA, tended to diminish with increasing filler concentration. These diminished mechanical properties are likely a consequence of the uneven dispersion of the filler, which is mainly governed by the random mixing of silica particles during the extrusion procedure itself [81]. In addition, the irregular shape of the filler particles can result in such inconsistency during the mixing of the particles, which can further contribute to the non-homogeneous dispersion of particles and result in stress concentrations at various positions along with the samples, resulting in mechanical failure [82]. The mechanical properties of the prepared samples are presented in Table 2. The arithmetic mean was used to calculate the SD values, as shown in Equation (1):

(1) $$\mathrm{SD}=\sqrt{\left(\sum X^{2}-n{\bar{X}}^{2}\right)/(n-1}),$$

where SD is the estimated standard deviation, X is the measured value of a single reading, n is the number of measurements, and $\bar{X}$ is the arithmetic mean of a set of observed measurement values. 

### 3.2. Thermal Characterization

**Figure 12:**
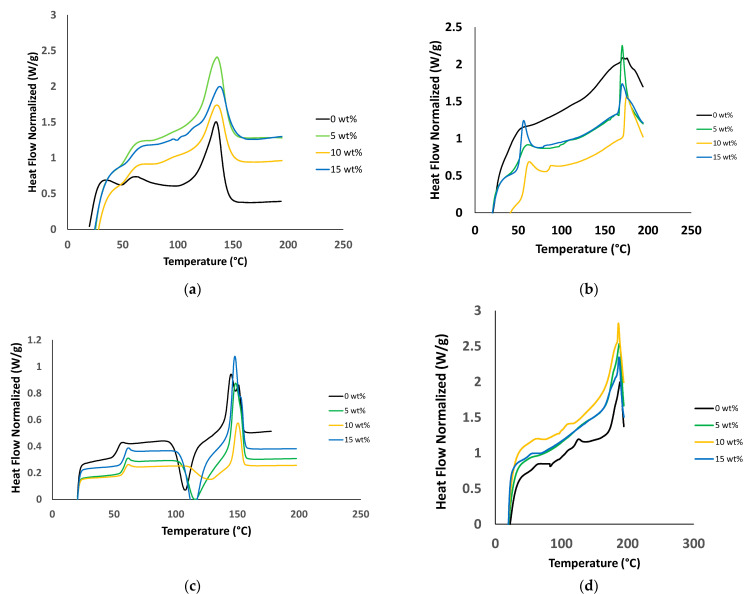
Melting peaks: (**a**) PP; (**b**) PVA; (**c**) PLA; and (**d**) Nylon.

DSC analysis was carried out at a heating rate of 10 °C/min on all the prepared sets of composites to elucidate their crystallization behaviours. Figure 12 shows the melting peaks (Tm) obtained from the DSC thermograms. The observed Tm for pure PP is 135 °C, which is slightly lower than the value reported for PP [5]. The Tm for pure PVA is 171 °C, which is in agreement with the value reported in the literature [84]. For PLA, two melting peaks were observed at 145 °C and 150 °C, consistent with the literature. These two peaks can be attributed to the coexistence of two PLA crystal structures known as the α and β forms. The α form is the peak at a higher value because of the better quality and size of crystals [84]. A sharp peak is observed at 189 °C in the thermogram of nylon [57]. A smooth transition temperature trend is observed for all the PLA samples, indicating that the pure PLA sample does not contain impurities. Furthermore, the addition of filler does not substantially change Tm. The slight variations in the shapes of the curves are likely associated with the degree of crystallinity in each sample [25], implying that the overall thermal stability of the composite material is maintained. 

The obtained DSC data were used to characterize the intrinsic thermal properties of the materials. For example, an increase in the filler concentration did not considerably affect the Tm, glass-transition temperatures (Tg), or crystallization temperatures (Tc) of the samples. Once the Tc, Tg, and Tm values are determined, the range of processing temperatures can be identified, providing a reasonable estimation of the suitability of the materials for industrial applications. The most common industrial processing temperature is ±40 °C [46,49]. In addition, when the Tc and Tm values of the materials are known, the enthalpies of the materials can be estimated, providing an understanding regarding how much energy and heat would be required in the manufacturing process for such composite materials, which is an important consideration from the industrial manufacturing perspective. The Tg value for PP ranged from 41 °C to 57 °C (reaching a maximum at 10 wt% filler concentration), which is slightly lower than the values reported elsewhere [5]. In the case of PVA and PLA, the Tg values ranged from 44 °C to 67 °C and from 52 °C to 58 °C, respectively, as reported for other PVA and PLA systems [84], with the highest Tg values being observed for the samples with 15 wt% silica in both the cases. In case of nylon, the Tg varied from 31 °C to 42 °C, with the highest value being obtained for the sample with 10 wt% silica. In any silica-containing sample, the Tg value of which increased compared with that of the pure polymer, silica particles may have prevented the thermal motion of the polymeric chains, resulting in the above phenomenon [27]. 

Slight variations in the Tc values and broadening and stretching during the crystallization process were observed in the case of samples containing silica. However, the added filler did not strongly affect the thermal characteristics of the composite materials, implying that they exhibit good thermal stability. The obtained results are consistent with previous studies involving various polymer/composite systems [85]. 

The areas under the cooling and heating curves were calculated to determine the enthalpies of crystallization and the melting enthalpies. The percent crystallinity was calculated as follows using Equation (2):

(2) $$X_{c}(\%)=\frac{\Delta H_{m}}{\Delta H_{100\%}\left(1-\theta \right)}\times 100\%$$

where Xc is the percentage crystallinity of the pure polymer, ∆Hm is the melting enthalpy, ∆H_100%_ is the melting enthalpy of a 100% crystalline polymer (170 J/g for PP [5], 67 J/g for PVA [25], 94 J/g for PLA [84], and 70 J/g for nylon [86]), and θ is the mass fraction of the filler (sand).

The crystallinity values were observed to fluctuate mainly between 13% and 35% for all the prepared samples, consistent with the results of several previous polymer composite studies [5,72,87]. Overall, one concentration in each set resulted in a high crystallinity. However, the specific concentration that exhibited the highest crystallinity percentage varied from one system to another. In all cases, the decreasing crystallinity may be caused by the formation of imperfect crystals in the polymer because of the presence of silica interfering in the polymeric chains owing to random dispersion and incompatibility [84]. In addition, this variation could be attributed to the insufficient amount of filler present at the surface, causing the agglomeration or accumulation of the filler at the interface, where a soft layer is formed that tends to impede the nucleating effect [21,88]. The opposite is true in the case of an increase in crystallinity, wherein the silica particles are speculated to induce a heterogeneous nucleation effect [5]. The fluctuating variation in thermal properties, such as Tc, ΔHm, and Xc, can also be linked to the conformational changes of the macromolecular structures during the crystallization process because the filler was spread randomly and not densely packed, thereby weakening the molecular interactions of the structures. Such behavior has been reported in several composite studies in which the thermal values do not substantially vary [72,88,89]. 

The Tg, Tc, Tm, enthalpies of crystallization (ΔHc), ΔHm, and Xc values for all the prepared composite materials are presented in Table 3.

### 3.3. SEM Characterization

**Figure 13:**
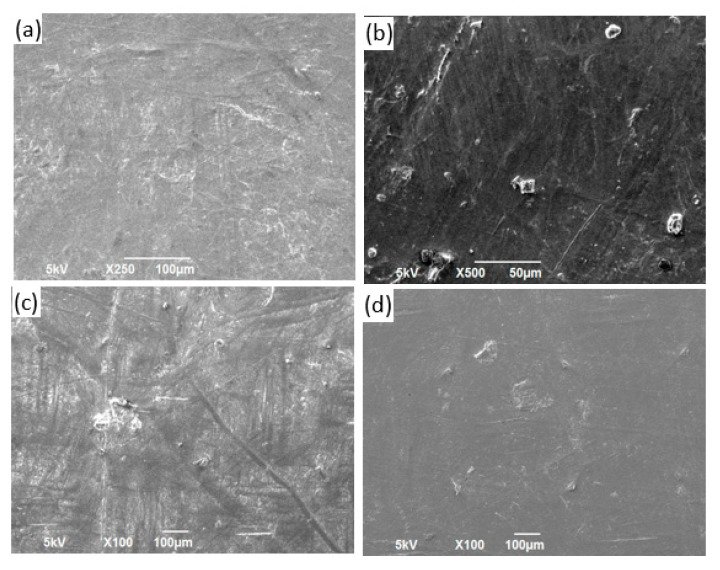
Images obtained using an SEM for PP/silica samples containing (**a**) 0 wt%, (**b**) 5 wt%, (**c**) 10 wt%, and (**d**) 15 wt% silica.

**Figure 14:**
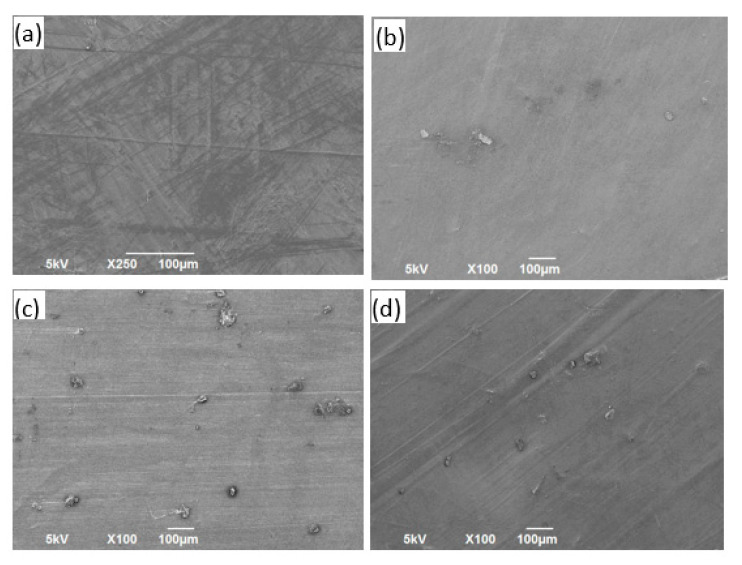
Images obtained using an SEM for PVA/silica samples containing (**a**) 0 wt%, (**b**) 5 wt%, (**c**) 10 wt%, and (**d**) 15 wt% silica.

**Figure 15:**
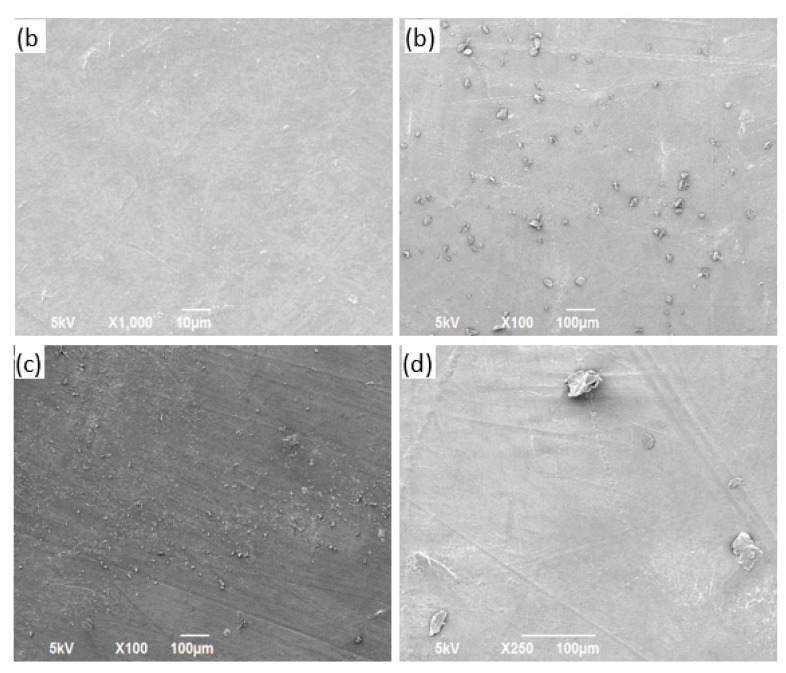
Images obtained using an SEM for PLA/silica samples containing (**a**) 0 wt%, (**b**) 5 wt%, (**c**) 10 wt%, and (**d**) 15 wt% silica.

**Figure 16:**
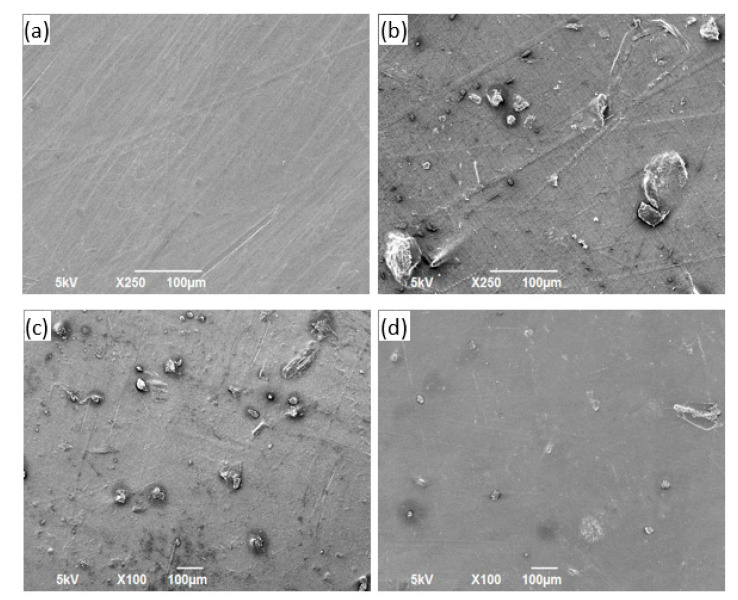
Images obtained using an SEM for Nylon/silica samples containing (**a**) 0 wt%, (**b**) 5 wt%, (**c**) 10 wt%, and (**d**) 15 wt% silica.

The morphologies of the neat polymer sheets and reinforced composite sheet materials are shown in the SEM images in Figure 13, Figure 14, Figure 15 and Figure 16 for PP, PVA, PLA, and nylon, respectively. Generally, a smooth surface was observed for all the neat polymer samples, consistent with several previous polymer/composite studies [8,21]. The SEM images of the silica reinforcements at concentrations ranging from 5 to 15 wt% confirm the random dispersion of silica particles in the corresponding matrices in all the prepared samples. The SEM images show that the silica particles are spherically shaped, as previously reported [1]. Moreover, the photos show that the particle density and extent of agglomeration increased with increasing filler concentration. When agglomeration begins, the mechanical and thermal properties are adversely affected, reflected in the experimental results reported in the literature [21]. 

In case of the PP/silica samples, a smooth surface is observed at 0 wt% silica. An increase in the number of randomly dispersed silica particles can be observed on the surface of the samples with 5 and 10 wt% silica, as evident in Figure 13b,c, respectively. Clumped particles are observed on the surface of the sample with 15 wt% silica (Figure 13d), indicating agglomeration. Similar behavior has been reported for PP/silica composites with similar silica loadings [90]. For PVA, increased dispersion is observed with increasing silica loading from 0 to 10 wt% (Figure 14a–c). For the samples prepared with PLA, good dispersion is observed at 5 and 10 wt% silica loadings (Figure 15b,c, respectively). The excellent distribution in these samples reflects the enhanced properties at these concentrations. At 15 wt% silica (Figure 15d), larger particles begin to appear at greater distances from each other. These particles correspond to agglomerates. A similar trend of random dispersion is also observed in the samples prepared from nylon (Figure 16). In addition, the SEM images indicate that in all of the samples, the particles are just settled in the matrix and not homogeneously mixed or well bonded to the matrix. The interaction and adhesion of the particles with the matrix are properties that require further study because they can strongly affect the enhanced mechanical and thermal behaviors of composite materials. 

### 3.4. Bulk Density

The procedure specified in ASTM D7263-09 was followed to determine the dry density of the silica filler used in this study [91]. The sand sample was dried using an electric drying oven and a controlled drying process. The density of the filler was calculated using Equation (3) [92]:

(3) $$\rho_{\mathrm{filler}}=\frac{m_{\mathrm{filler}}}{V}$$

where $\rho_{\mathrm{filler}}$ is the density of the filler in kg/m^3^, m_filler_ is the mass of the filler in kg, and *V* is the volume of the measuring cylinder in m^3^.

The apparent densities of the polymers were determined according to ASTM D792-20 [93]. After the masses of the samples in air and water were measured, their corresponding apparent densities can be calculated using Equation (4) [92]:

(4) $$\rho_{\mathrm{polymer}}=\frac{m_{\mathrm{air}}\;\rho_{\mathrm{water}}}{m_{\mathrm{air}}+{\;m}_{\mathrm{water}}}$$

where *ρ*_polymer_ is the apparent density of polymer in kg/m^3^, m_air_ is the mass of the sample measured in air in kg, m_water_ is the mass of the sample measured in water in kg, and *ρ*_water_ is the density of water in kg/m^3^. 

To estimate the density of the composite material, the theoretical density of the composite material was first estimated using the linear rule of mixing, which assumes that the composite property is the volume-weighted average of the matrix and dispersed phases. This equation also assumes that there is no inclusion of air in the composite material and that the filler does not affect the density of the polymeric matrix by inducing nucleation or crystal formation. Equation (5) was used to calculate the density of the composite material [94]:

(5) $$\rho_{c}=\frac{\rho_{\mathrm{filler}}\;\rho_{\mathrm{matrix}}}{\rho_{\mathrm{matrix}}{\;m}_{\mathrm{filler}\;}+\rho_{\mathrm{filler}}\;(1-m_{\mathrm{filler}})}$$

where *ρ*_c_ is the density of the composite in kg/m^3^, *ρ*_filler_ is the density of the filler (silica) in kg/m^3^, *ρ*_matrix_ is the density of the polymer matrix (i.e., PP, PVA, PLA, and nylon) in kg/m^3^, and m_filler_ is the mass fraction of the filler in kg. 

A precise weighing balance (Citizen-CX 220, d 0.0001 g, CITIZEN SCALE PVT. LTD, Mumbai, India) was used to measure the weight of the composite materials. For the volume measurements, the prepared samples were cut with a cutter according to ASTM D6287-17 [95]. The thickness of each sample was measured along five different points using a Mitutoyo thickness gage (model 547-526S) to ensure uniformity. The data collected from the weighing balance and thickness gage were used to estimate the experimental bulk densities of the samples by calculating their corresponding mass-to-volume ratios. 

## 4. Discussion

The selected samples were subjected to tensile testing in accordance with ASTM 638. The tensile strength (TS) of the prepared samples was calculated by dividing the maximum applied load with the average cross-sectional area of the gage length of the specimen, as shown in Equation (6):

(6) $$\mathrm{TS}\;\left[\mathrm{MPa}\right]=\frac{F_{\max}}{A}$$

where TS is the tensile strength in MPa, F_max_ is the maximum force exerted on the specimen under tension in N, and *A* is the average cross-sectional area of the sample in mm^2^.

Further, the elastic modulus was estimated by extending the linearity of the load–extension plot and dividing the difference between the stress values at any given corresponding point of the specified segment on this line with its corresponding difference in strain values according to Equation (7). In addition, the modulus of elasticity was calculated based on the average initial cross-sectional area concerning the gage length. However, for any sample for which no proportionality was observed, the corresponding secant value was estimated by forming a tangent line and noting the respective strain value from the yield point at which the tangent passes through the zero-stress value. The individual stress value used in the computation was then defined by dividing the initial load–extension curve with the actual average cross-sectional area of the prepared sample:

(7) $$E\;\left[\mathrm{MPa}\right]=\frac{F\times L_{\mathrm{original}\;}}{A\times \Delta L}$$

where E is the elastic modulus in MPa, F is the force exerted on the specimen under tension in N, L_original_ is the original gauge length of the sample in mm, A is the original cross-sectional area of the specimen in mm^2^, and ∆L is the change in length of the specimen in mm.

Elongation is useful when considering uniform deformation with respect to the gage length of the sample. Elongation values are quantitatively crucial for realizing good engineering designs. In addition, when the sample undergoes necking (nonuniform deformation) along the gage length, the corresponding nominal strain values are measured. Further, based on the observed elongation, the extension of the sample at the final point of rupture can be divided with the initial gage length and multiplied by 100 to prevent stretching at break (strain at failure or ductility). 

The ductility of the samples can be calculated as follows:

(8) $$\mathrm{Ductility}\;\left(\%\right)=\frac{L_{\mathrm{final}}-L_{\mathrm{original}\;}}{L_{\mathrm{original}}}\times 100$$

where L_original_ is the original gage length of the specimen in mm and L_final_ is the final length at break of the specimen in mm.

The toughness of a material indicates the amount of energy that it can absorb on impact and can be estimated by determining the area under the stress-strain plot. The toughness can be calculated as follows:

(9) Toughness [MPa]=σ×ε

where σ is the stress in N and ε is the strain.

The yield stress values of the specimens can be determined by marking the intersection point of the 0.2% offset stress values in the original stress–strain plot. 

Through this study, the significant impact of silica on different polymeric waste was established and studied. Furthermore, when silica was added to polymeric waste, the mechanical, thermal, and bulk density properties of the material can be altered according to the application requirements [30]. Herein, composite sheets with different polymers blended with various amounts of silica were successfully prepared via melt blending and hot compression molding. The TS of the developed composite materials increased with increasing filler concentration to 5 wt% in the case of PVA. In contrast, in the case of PP/silica and PLA/silica samples, the peak values corresponded to 10 wt% filler. This higher optimum filler content reflects the enhanced mechanical properties imparted by silica reinforcement. In all the developed samples, the values of other mechanical properties (e.g., toughness, ductility, elastic modulus, and yield stress) decreased when the filler content exceeded a certain level, indicating a weakening of the matrix owing to agglomeration. Among the thermal properties, the melting temperatures and glass-transition temperatures did not substantially vary, promoting the thermal stability of the developed composite material with increasing filler addition. Moreover, the crystallinity varied from 14% to 35% in all cases, corresponding to the random dispersion of the particles at various weight percentages of silica. SEM analysis showed that the particles are dispersed randomly and non homogeneously, which is a property that must be improved to further enhance the properties of the composites. For the bulk density, a decreasing trend was observed in all the polymeric samples with an increasing concentration of silica particles. This trend is advantageous for industrial applications because a low density facilitates the easy handling and transportation of composite materials.

**Figure 17:**
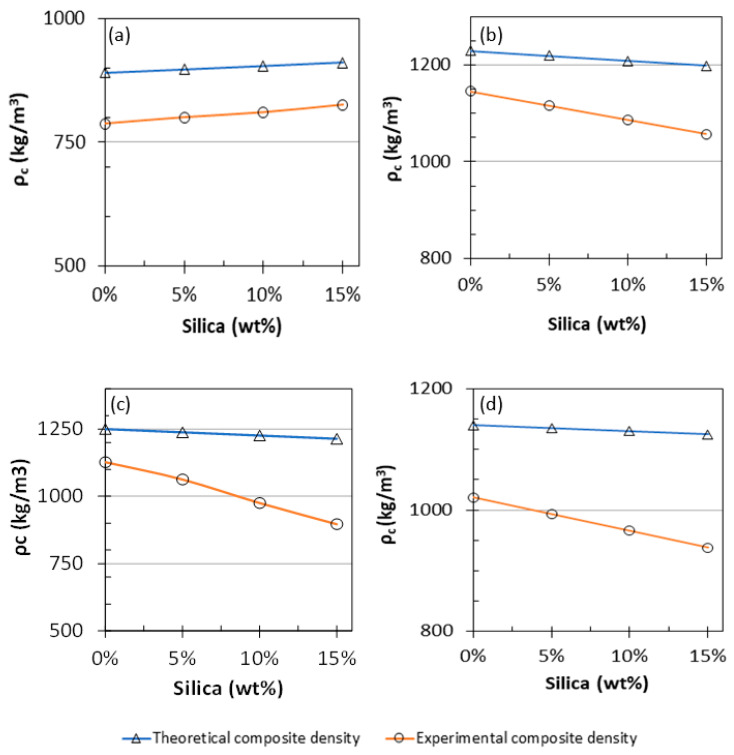
Theoretical and experimental composite bulk densities of (**a**) PP/silica, (**b**) PVA/silica, (**c**) PLA/silica, and (**d**) nylon/silica composites.

Generally, the composite density decreased with the increasing filler concentration, except in case of the PP theoretical data, which indicated the opposite behavior. This may be linked to be the fact that the density of the filler (1047 kg/m^3^) was lower than that of the polymers. The case in which the density increased with increasing filler concentration is explained by the dispersion of the filler. In the case of PP, the density varied from 788 to 826 kg/m^3^ as the filler concentration was varied from 0 to 15 wt%, respectively. In the PVA samples, the density decreased from 1142 kg/m^3^ to 1057 kg/m^3^ as the filler content was increased from 0 to 15 wt%, respectively. For PLA, other researchers have reported a steady decreasing density trend, including a 9.8% decrease at 0 wt% and 26.2% decrease at 15 wt% [67]. Similarly, for nylon, a decrease of 10% was observed at 0 wt% along with a reduction of 17% at 15 wt%. Such variations are speculated to be caused by formation of air pockets during the process attributed to the the high temperatures applied when processing the samples that would affect the density. In addition, because of poor mixing, residual moisture can be degassed within the material during the processing phase, further decreasing the material’s density because of the high viscosities achieved by the polymeric matrix [94,96]. From an industrial perspective, using fillers to decrease the densities of polymers is potentially helpful in developing composite materials that float [97]. Furthermore, decreasing the densities for such composite materials enables the material to be handled more efficiently, which can be considered as an essential design parameter to reduce the costs involved with transportation [98]. Table 4 presents the theoretical and experimental data for bulk densities, and Figure 17 shows a comparison between the theoretical and practical densities of all the prepared composite materials. The properties observed in this study confirm the effect of silica as a filler. Further experiments are underway to study the same systems using a suitable compatibilizer to characterize the effect of an additional agent on the behavior of the composite materials, which could potentially enhance the interaction and integrity of the filler–polymer matrices. Such a mechanochemical study would aid in understand the 3D printing capability of such materials and expand the possibility of reusing the silica/waste polymer materials in 3D printing technology [31,99] and to avoid the possible failure mechanism that could be caused by 3D Printing [100].

## 5. Conclusions

In general, the tensile strength is improved due to mixing silica into the polymeric waste from 3D printing. For PLA, this increase was up to 10%, whereas the impact on PP mixtures was insignificant, on the contrary the increase in the strength to 5% was observed for PVA and nylon. On the other hand, for all polymeric mixtures, the toughness dropped due to the increase silica percentage, and a similar observation was concluded for the ductility. Moreover, a noticeable decrease in the modulus of the elasticity was observed as the silica percentages increased, and the same trend was observed for the yield strength, except for the PLA. A consistent decrease in the density of the silica/polymers mixtures was observed more silica was added, except for the PP that showed a slight increase.

In general, it has been reported that recycling polymeric waste of the 3D printing process will affect different aspects of the properties of the 3D printed parts like shear stress, temperature, and oxygen contents that occur during extrusion and degrade extruded polymers [102]. Moreover, the process takes place not only in polymers sensitive to these factors but also in polymers that are relatively resistant [100] in addition to the fact the changes in the physical properties of the polymer significantly influence the manufacture of high-quality extrusion products. It has been found that multiple extrusiona of polymers have a strong influence on their change in viscosity, molecular weight, and breaking strength, where changes in properties are generated by factors such as temperature, but also by the amount of extrusion of one material [103]. Ultimately, additive manufacturing becoming a valuable tool for many industries as equivalent and subtractive manufacturing processes [104].

## Figures and Tables

**Table 1:** Dimensions of the prepared tensile test specimens.

Polymer Type	Dimensions (l × w × t) (mm)			
	0 wt%	5 wt%	10 wt%	15 wt%
PLA	10 × 4 × 0.06	10 × 4 × 0.06	10 × 4 × 0.06	10 × 4 × 0.08
PP	10 × 4 × 0.1	10 × 4 × 0.1	10 × 4 × 0.12	10 × 4 × 0.12
PVA	10 × 4 × 0.06	10 × 4 × 0.08	10 × 4 × 0.12	10 × 4 × 0.12
Nylon	10 × 4 × 0.04	10 × 4 × 0.06	10 × 4 × 0.1	10 × 4 × 0.1

**Table 2:** Mechanical properties of all the prepared composite samples.

PP/silica	Tensile Strength (MPa)	SD	Toughness (MPa)	SD	Ductility (%)	SD	Elastic Modulus (MPa)	SD	Yield Stress (MPa)	SD
100:0	11	±1	95	±18	947	±148	72	±13	6	±0.5
95:5	7	±1	33	±27	147	±65	58	±11	5	±0.5
90:10	7	±1	8	±4	104	±32	53	±10	6	±0.9
85:15	6	±0.8	6	±2	96	±26	41	±3	5	±0.6
PVA/silica										
100:0	54	±15	11	±8	26	±16	995	±28	16	±4
95:5	76	±7	5	±0.7	14	±0.6	871	±5	11	±2
90:10	30	±13	2	±0.5	13	±3	540	±76	9	±0.3
85:15	31	±12	2	±1	11	±5	167	±112	10	±2
PLA/silica										
100:0	63	±7	4	±1	11	±3	896	±86	53	±20
95:5	67	±4	5	±1	13	±2	1020	±82	63	±1
90:10	121	±13	6	±1	15	±1	840	±79	64	±12
85:15	54	±8	3	±0.4	11	±0.1	793	±95	53	±9
Nylon/silica										
100:0	120	±26	259	±30.60	239	±33	784.43	±148	71	±12
95:5	46	±12	148.22	±39	220	±56	554.95	±27	35	±14
90:10	26	±9	85.58	±36	243	±26	262.64	±51	23	±7
85:15	23	±6	50.38	±14	188	±30.45	257	±30	22	±1

**Table 3:** Thermal properties of all the prepared samples.

PP/silica	Tg (°C)	Tc (°C)	Tm (°C)	ΔHc (J/g)	ΔHm (J/g)	Xc (%)
100:0	54.1	89.1	134.8	39.2	27.7	16.3
95:5	55.0	89.2	135.4	50.0	44.0	25.9
90:10	57.0	89.0	135.4	5.7	24.2	14.2
85:15	41.1	88.9	138.0	29.6	24.1	14.1
PVA/silica						
100:0	43.6	136.3	170.6	15.7	23.2	34.6
95:5	52.0	135.5	170.1	10.2	14.0	20.9
90:10	58.0	133.0	175.3	13.4	12.8	19.1
85:15	66.8	135.1	170.0	12.8	19.2	28.6
PLA/silica						
100:0	52.1	107.9	144.9	33.0	25.6	27.3
95:5	57.6	116.4	148.6	35.4	30.0	31.9
90:10	57.0	128.1	150.4	15.1	13.7	14.5
85:15	57.7	115.1	147.9	49.9	31.3	33.3
Nylon/silica						
100:0	31.1	149.4	188.6	56.1	11.1	15.8
95:5	36.9	150.1	187.7	20.1	19.4	27.7
90:10	41.6	151.0	186.1	27.4	17.5	24.9
85:15	36.4	151.6	187.1	27.6	18.6	26.6

**Table 4:** Theoretical and experimental composite bulk densities for all the prepared samples.

PP/silica	Theoretical Density (kg/m^3^)	Experimental Density (kg/m^3^)
100:0	1050 [101]	787.50
95:5	1049.88	800.05
90:10	1049.75	810.42
85:15	1499.24	825.50
PVA/silica		
100:0	1290 [98]	1141.90
95:5	1275.24	1116.32
90:10	1260.82	1086.74
85:15	1755.77	1057.16
PLA/silica		
100:0	1250 [83]	1126.89
95:5	1238.04	1062.38
90:10	1226.30	974.98
85:15	1714.70	896.00
Nylon/silica		
100:0	1350 [98]	1020.50
95:5	1330.79	992.94
90:10	1312.11	965.37
85:15	1816.20	937.81

## Data Availability

Not applicable.
